# Recombinant Expression of a Novel Fungal Immunomodulatory Protein with Human Tumor Cell Antiproliferative Activity from *Nectria haematococca*

**DOI:** 10.3390/ijms151017751

**Published:** 2014-09-30

**Authors:** Shuying Li, Ying Nie, Yang Ding, Lijun Shi, Xuanming Tang

**Affiliations:** 1 Institute of Agro-products Processing Science and Technology, Chinese Academy of Agricultural Sciences (CAAS), Key Laboratory of Agro-products Processing, Ministry of Agriculture, No. 2 Yuan Ming Yuan West Road, Beijing 100193, China; E-Mails: lishuying2000@163.com (S.L.); nieying@caas.cn (Y.N.); dingyang@caas.cn (Y.D.); 2 Institute of Animal Science and Veterinary Medicine, CAAS, No. 2 Yuan Ming Yuan West Road, Beijing 100193, China; E-Mail: shilijun@caas.cn

**Keywords:** antitumor, fungal immunomodulatory protein (FIP), hemagglutination, interleukin-2 (IL-2), *Nectria haematococca*, proliferation

## Abstract

To our best knowledge, all of the fungal immunomodulatory proteins (FIPs) have been successfully extracted and identified in Basidomycetes, with only the exception of FIP from ascomycete *Nectria haematococca* (FIP-nha) discovered through homology alignment most recently. In this work, a gene encoding FIP-nha was synthesized and recombinantly expressed in an *Escherichia coli* expression system. SDS-PAGE and MALDI-MS analyses of recombinant FIP-nha (rFIP-nha) indicated that the gene was successfully expressed. The yield of the bioactive FIP-nha protein was 42.7 mg/L. *In vitro* assays of biological activity indicated that the rFIP-nha caused hemagglutination of human and rabbit red blood cells, significantly stimulated mouse spleen lymphocyte proliferation, and enhanced expression of interleukin-2 (IL-2) released from mouse splenocytes, revealing a strong antitumor effect against HL60, HepG2 and MGC823. Through this work, we constructed a rapid and efficient method of FIP production, and suggested that FIP-nha is a valuable candidate for use in future medical care and pharmaceutical products.

## 1. Introduction

Medicinal mushrooms are widely used to treat malignancies in folk herbal therapies [1]. Many bioactive components have been identified from Basidiomycete mushrooms, including polysaccharides [2], triterpenes [3], and fungal immunomodulatory proteins (FIPs) [4]. FIPs belong to a new protein family with high sequence and structural similarities [5,6]. Known FIPs have high sequence identities (57%–100%), consist of 111–114 residues with molecular weights of 12.4–15.0 kDa, and lack cysteine, histidine and methionine residues [4,5,6]. Crystal structure analysis indicates that FIPs are non-covalently linked homodimers, consisting of an *N*-terminal dimerization domain and a *C*-terminal fibronectin III (FNIII) domain [7,8].

Despite being highly conserved in primary sequence and structure, FIPs family members have disparate biological activities [8]. Aggregation of FIPs is commonly observed in red blood cells, and different FIPs uniquely hemagglutinate various red blood cells [4,5]. FIPs can stimulate the proliferation of mouse splenocytes [9] and human peripheral blood lymphocytes (hPBLs) [10,11], leading to expression changes in several cytokines including interleukin-2 (IL-2), interferon-γ (IFN-γ), and tumor necrosis factor-α [12]. *In vivo*, FIPs can also prevent systemic anaphylactic reactions and significantly decrease mouse footpad edema during the Arthus reaction [13]. In addition to immunomodulatory activities, antitumor functions of FIPs have been researched extensively [14,15]. Thus far, FIPs appear to be promising for the treatment of various human diseases.

Since the first FIP was isolated from *Ganoderma lucidum* (LZ-8) in 1989 [5], only ten other FIPs have been isolated and purified from *Ganoderma* spp. (FIP-gts, FIP-gja, and FIP-gmi) [16,17], *Flammulina velutipes* (FIP-fve) [4], *Volvariella volvacea* (FIP-vvo) [6], *Poria cocos* (FIP-pcp) [18], and *Trametes versicolor* (FIP-tvc) [19]. Purification of FIPs from wild or cultivated mushrooms remains low yielded, complicated, time consuming, and costly, so research is now focused on identifying new FIPs by other molecular methods and characterizing their expression. Aim to obtain sufficient FIPs for practical applications, many genetic engineering approaches have been performed to improve the yields of FIPs. So far, the most developed expression system is using *E. coli*, yeast and insect cells. The gene encoding FIPs has been effectively expressed in these prokaryotes and eukaryotes expression systems [20]. Nowadays the yeast and insect cell expression systems are commonly used in FIPs production, with regards to the proper folding and modification after transcription, especially the glycosylation. However, these two expression systems are also faced with the disadvantages of long growth cycle, high cost, and complicated purification procedure. Unlike the expression of FIPs with a His tag mainly insoluble in *E. coli*, the FIP recombinant proteins produced in *E. coli* with a GST tag are soluble and reach a high quantity, which can be convenient for their future industrial applications [21].

FIP from *Nectria haematococca* (FIP-nha), the latest reported novel FIP, was identified in the ascomycete *Nectria haematococca* using homology alignment [22] and represents a new FIP discovered beyond Basidiomycota. The expression of recombinant FIP-nha (rFIP-nha) in a yeast expression system [22] has been studied; however, the yield of this system is insufficient for massive production. Moreover, the systematic function studies and clinical applications of FIP-nha have rarely been reported. Hence, construction of large-scale production system and detection of activities *in vitro* are indispensable for the practical application of this promising immune modulator. In this study, we first successfully expressed FIP-nha in an *E. coli* expression system with a GST tag, and investigated its immunomodulatory and antitumor activities *in vitro*.

## 2. Results and Discussion

### 2.1. Expression, Purification and Identification of rFIP-nha

**Figure 1:**
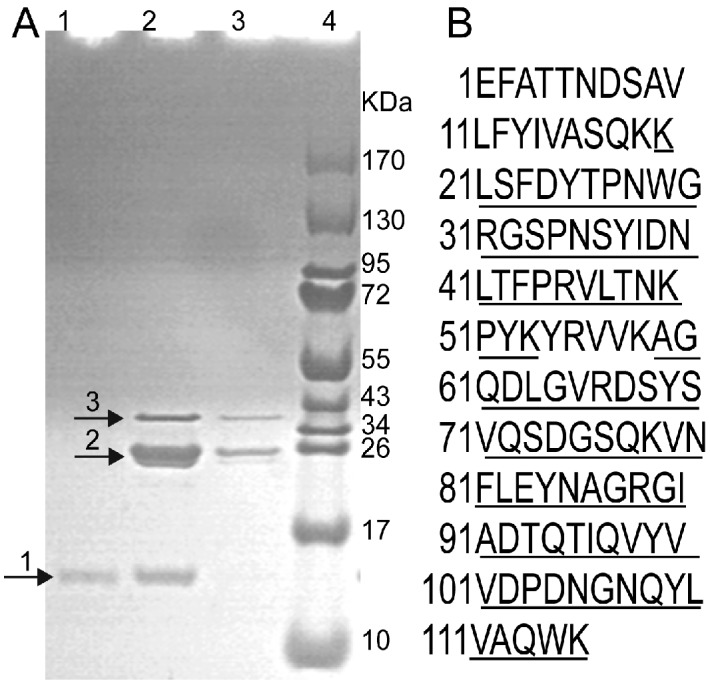
Purification and identification of rFIP-nha. (**A**) Tricine-SDS-PAGE analysis. GST-FIP-nha was purified using a GST column, digested with thrombin and eluted with PBS and glutathione. Lane 1: Eluate with PBS. Lanes 2 and 3: Eluates with glutathione. Lane 4: Standard protein molecular weight markers. Arrows 1, 2, and 3 indicated the purified rFIP-nha, GST, and GST-FIP-nha, respectively; (**B**) Amino acid sequence of deduced FIP-nha. Nine peptide fragments consisting of 91 residues identified by MALDI-MS are underlined.

The deduced FIP-nha consists of 114 residues (calculated molecular weight = 12,837 Da), lacking cysteine, histidine and methionine residues, which are typical of the FIPs family. FIP-nha is 67% identical to FIP-gmi, 66% identical to LZ-8, 64% identical to FIP-gja, 63% identical to FIP-vvo, and 59% identical to FIP-fve. The gene for FIP-nha was custom-synthesized and expressed in *E. coli* as a GST fusion protein. The fusion protein GST-FIP-nha was soluble when induced at 25 °C with 0.1 mM IPTG. A protein band (~39 kDa) was observed with Tricine-SDS-PAGE (Figure 1), a finding that agreed with the calculated molecular weight of GST-FIP-nha (38.837 KDa). The product was purified with a GST column, and the fusion protein was digested with thrombin to release rFIP-nha with the expected size of 13 kDa (Figure 1). Approximately 42.7 mg of rFIP-nha protein was obtained from 1 L of cultures. This quantity of rFIP-nha protein was sufficient for bioactivity analysis and future industrial applications. To confirm protein identity, proteins were analyzed with MALDI-MS. Nine peptide fragments were obtained, corresponding to 91 residues of deduced FIP-nha (77% coverage; Figure 1). This result indicated that the purified protein was indeed rFIP-nha. To our knowledge, this is the first successful heterologous expression and production of FIP-nha in an *E.coli* expression system to be reported.

Recombinant protein of FIP-nha has been produced in yeast expression system with a yield of 18.9 mg/L [22]. However, improving the production rate and simplifying the purification procedure are important. The *E. coli* expression system is a widely used expression platform because of its advantages of short growth cycle, low cost and high yield [23]. In this study, bioactive FIP-nha production in *E. coli* reached 42.7 mg/L, which was two times higher than the result of Bastiaan-Net *et al.* in 2013 [22]. In addition, GST fusion expression greatly simplified the purification procedure of small molecular weight rFIP-nha.

### 2.2. Hemagglutination Activity of rFIP-nha

We used all four types of human blood (A, B, AB, and O) and rabbit red blood cells to measure hemagglutination activity of rFIP-nha. RFIP-nha weakly hemagglutinated human blood cells and the hemagglutinating concentrations for four human erythrocytes exceeded 100 µg/mL. Meanwhile rFIP-nha strongly hemagglutinated rabbit blood cells (minimal concentration = 1.275 µg/mL). It has been reported that rFIP-nha, produced by the *Pichia pastoris* expression system, had hemagglutinating activity with rabbit blood cells at concentrations greater than 5 µg/mL [22]. Our data show that biological activity of rFIP-nha expressed in *E. coli* was not compromised by this prokaryotic system.

Agglutinating activity to red blood cells was always used to monitor FIPs during the purification process. FIPs have various degrees of agglutinating activity toward different types of red blood cells [4,5,6,22,24,25]. LZ-8 was reported to aggregate sheep and mouse red blood cells [5] and FIP-vvo agglutinated rat, mouse and rabbit red blood cells [6]. Except for FIP-fve, no aggregation activity was observed with human red blood cells [4]. RFIP-nha had obvious agglutinating activity in rabbit red blood cells, but weaker activity in all four human red blood cell types. FIPs lacking agglutinating activity to human red blood cells may offer promise for therapeutic use in the future [5].

### 2.3. The Immunomodulatory Activity Assay of rFIP-nha

In a mouse splenocyte proliferation assay, as shown in Table 1, the effects of rFIP-nha on lymphocyte proliferation were observed when combined with a T-cell mitogen, ConA, or a B-cell mitogen, LPS. ConA- or LPS-induced splenocyte proliferation was significantly enhanced by rFIP-nha (at 2 and 4 μg/mL). When lymphocytes were incubated without ConA or LPS, rFIP-nha still significantly stimulated lymphocyte proliferation (at 1, 2, 4, 8, 16, and 32 μg/mL). The stimulatory effect of rFIP-nha on lymphocyte proliferation, with or without mitogens, was strongly depends on the concentrations of rFIP-nha, and had a bell-shaped dose-response curve. Maximal stimulatory effects occurred at 2 μg/mL, which was significantly different from the control groups (Table 1). No synergistic effect was observed when both rFIP-nha and ConA or LPS were supplemented. Lymphocytes are key immune effector cells and the above data indicate that different subpopulations of lymphocytes were variously activated by rFIP-nha.

**Table 1:** Effects of rFIP-nha on lymphocyte proliferation (expressed at 570 nm). Values represent mean ± SD (*n* = 3). a: *p* < 0.05; b: *p* < 0.01 *vs.* control.

Sample	Concentration (μg/mL)	PBS (A_570_)	ConA (A_570_)	LPS (A_570_)
Control	-	0.16 ± 0.01	0.33 ± 0.03	0.32 ± 0.01
rFIP-nha	1	0.26 ± 0.02 ^a^	0.35 ± 0.01	0.38 ± 0.02
	2	0.33 ± 0.02 ^b^	0.41 ± 0.00 ^a^	0.43 ± 0.00 ^a^
	4	0.31 ± 0.01 ^b^	0.40 ± 0.00 ^a^	0.40 ± 0.01 ^a^
	8	0.25 ± 0.00 ^a^	0.35 ± 0.00	0.35 ± 0.01
	16	0.25 ± 0.00 ^a^	0.34 ± 0.00	0.34 ± 0.00
	32	0.24 ± 0.01 ^a^	0.33 ± 0.01	0.33 ± 0.02

**Figure 2:**
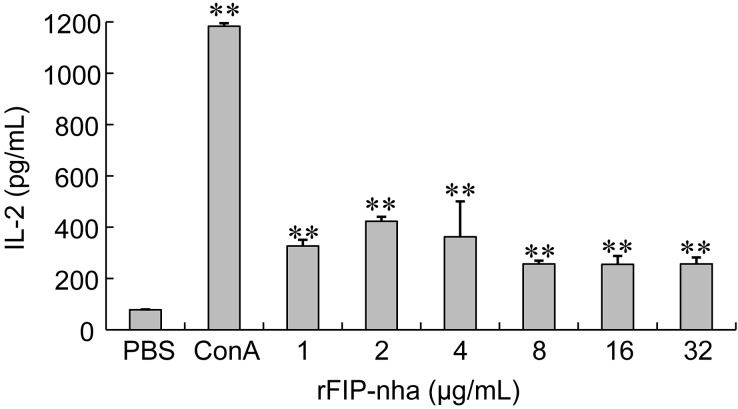
Effect of rFIP-nha on IL-2 release from mouse splenocytes. RFIP-nha (1, 2, 4, 8, 16, or 32 µg/mL) was used to treat mouse splenocytes for 48 h. Each bar represents mean ± SD (*n* = 3). ConA: positive control; PBS: negative control. **: *p* < 0.01, compared to negative control.

The immunomodulatory activity of rFIP-nha was estimated by analyzing its ability to induce IL-2 release from murine splenocytes. ConA, known to induce cytokine production in lymphocytes, was used as a positive control [26]. Data show that IL-2 released from mouse splenocytes followed a bell-shaped dose-response curve with respect to rFIP-nha concentrations (Figure 2), similar to those of other FIPs [4,11]. IL-2 produced with 1, 2, 4, 8, 16, or 32 µg/mL rFIP-nha was significantly different from controls (*p* < 0.01). Maximal stimulation was achieved at 2 μg/mL (IL-2 release = 423.17 pg/mL). IL-2 has been reported to be induced by 10 µg/ml of rFIP-gts or rFIP-fve purified from *E. coli* (412.8 and 165.3 pg/mL, respectively) [27,28]. The positive control, ConA (5 µg/mL), clearly enhanced IL-2 expression (1183.58 pg/mL) and this fell within the range of 971.2–1498.5 pg/mL [26].

Previous studies also showed that natural and recombinant FIPs stimulated the proliferations of human peripheral blood mononuclear cells (PBMCs) and murine splenocytes with increased cytokine productions [4,27,29,30,31,32]. It has been demonstrated that all FIPs from *Ganoderma* spp. and *F. velutipe* skewed the response to Th1 cytokine IL-2 and IFN-γ productions [29,30,31,32], but only FIP-vvo from *V. volvacea* skewed the response to Th2 cytokine IL-4 production [6]. The obtained rFIP-nha in this study increased the secretion of IL-2 at suitable concentrations, which seems to indicate that the immunomodulatory activity of rFIP-nha was more similar to those of FIPs from *Ganoderma* spp. and *F. velutipe* than that of FIP-vvo. However, more kinds of cytokine production and intensive research need to be conducted to elucidate the immune modulatory mechanism of rFIP-nha.

### 2.4. The Antitumor Activity Assay of rFIP-nha on Human Tumor Cells

**Figure 3:**
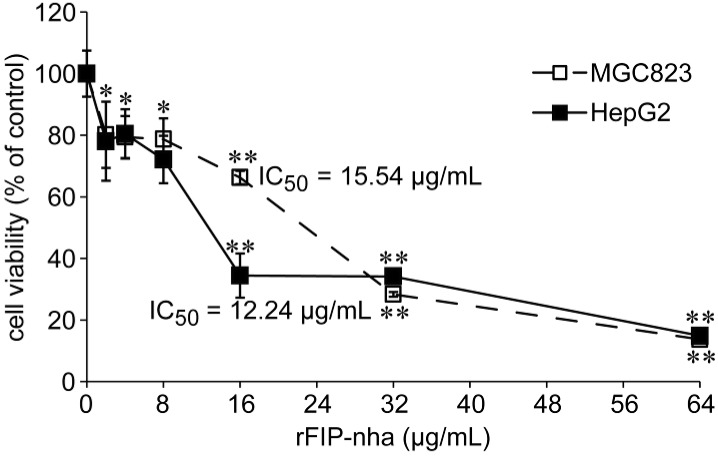
Proliferation inhibitory effects of rFIP-nha on MGC-823 and HepG2 cells measured by MTT assay. Data are presented as mean ± SD (*n* = 3). * and ** indicate *p* < 0.05 and *p* < 0.01 compared to the negative control.

The inhibitory effects of rFIP-nha were tested on human tumor cell gastric cancer MGC823 and liver cancer HepG2 cell lines at different concentrations (2, 4, 8, 16, 32, and 64 µg/mL) *in vitro* by MTT assay. RFIP-nha had significant antiproliferative effects on both tumor cells at 16, 32 and 64 µg/mL (*p* < 0.01) (Figure 3). Treatment of MGC823 and HepG2 with 16 µg/mL rFIP-nha for 24 h resulted in inhibition by 34% and 66%, respectively. When treated with 64 µg/mL rFIP-nha for 24 h, both cells lines were inhibited by up to 86%. Based on OD values, IC_50_ values of rFIP-nha in two tumor cell lines were 15.54 and 12.24 µg/mL, respectively (Figure 3). Data show that rFIP-nha was a potent tumor inhibitor in these cell lines, especially with HepG2.

**Figure 4:**
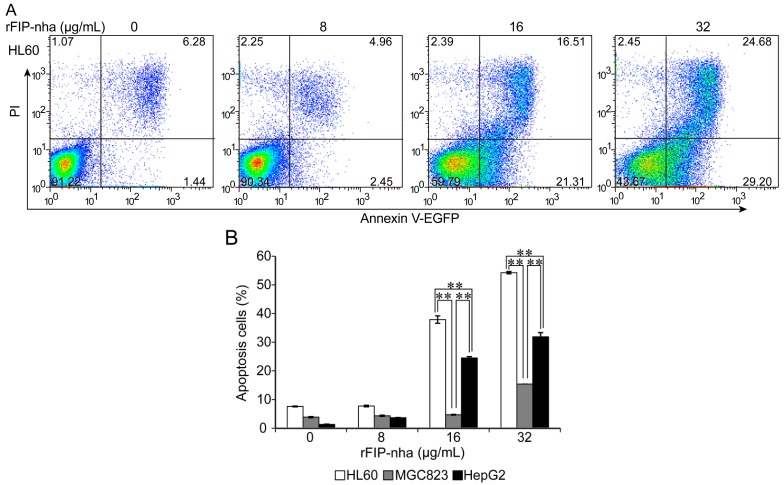
Apoptotic effects of rFIP-nha on human tumor cells. (**A**) RFIP-nha (8, 16, or 32 µg/mL) was incubated with HL60 cells for 24 h. Apoptotic induction was determined by flow cytometric analysis of Annexin V-EGFP and PI-staining. The upper right quadrant (UR) represented late apoptotic cells stained with Annexin V-EGFP and PI, and the lower right quadrant (LR) represented early apoptotic cells stained with Annexin V-EGFP; (**B**) Phase percentages for UR quadrant and LR quadrant are depicted on bar graph. Each bar represents mean ± SD (*n* = 3). **: *p* < 0.01.

Flow cytometric analyses of Annexin V-EGFP and PI-staining were used to determine whether rFIP-nha increased the apoptosis of the HL60, MGC823 and HepG2 tumor cells. Apoptosis was observed at 24 h, and apoptotic cells in the lower right quadrant (LR: Annexin^+^ PI^−^) and upper right quadrant (UR: Annexin^+^ PI^+^) regions were counted. As shown in Figure 4, rFIP-nha had significant apoptotic effects at 16 and 32 µg/mL. Proportions of apoptotic cells at these two doses were 37.89% and 54.25% for HL60, 4.69% and 15.42% for MGC823, and 24.50% and 31.91% for HepG2, respectively. Data show that rFIP-nha could effectively induce HL60, MGC823, and HepG2 tumor cell apoptosis in a dose-dependent manner, and that apoptotic effects were cell specific: the most potent apoptotic effects was observed in HL60 cells (followed by HepG2 and MGC823 cells).

Previous studies demonstrated that rLZ-8 and rFIP-gts inhibited cell proliferation by increasing G1 arrest [33,34,35,36], rFIP-fve suppressed A549 cell proliferation via the p53 activation pathway [37] and FIP-gmi inhibited metastatic ability regulated by epidermal growth factor in A549 cells [38]. These observations suggest that FIPs can be therapeutic. Suppression of tumor cell growth is associated with an increase in polyfunctional T cells that secrete multiple effector cytokines, such as IL-2, IFN-γ and IL-17 [39,40]. The potential mechanism for rFIP-nha induced apoptotic effects needs to be further explored.

FIPs are classified into a distinct family based on the amino acid sequence and biological activity similarity. A lot of factors influence the protein activities, such as proper folding, and post-transcriptional modification. Carbohydrate modification used to be considered as a key factor for keeping biological activity of FIPs. However, literature results and our study all contradict this hypothesis. The natural FIPs except LZ-8 were all found to lack carbohydrate modifications [4,41]. The activity of FIP-gts was lower when expressed in *E. coli* than in natural fruiting bodies [16]. On the other hand, the activity was lightly influenced when FIP-gts without glycosylation was expressed in insect cells [42]. RLZ-8 and rLZ-9 without glycosylation were functionally expressed in yeast cells [22]. FIP-tvc expressed in *E. coli* had significant biological activities [19]. FIP-fve was successfully and functionally expressed in *E. coli* with a His-tag [43], but when FIP-fve was expressed in *E. coli* with a GST tag, only 50% activity of natural FIP-fve was left [44]. To our knowledge, proteins expressed in *E. coli*, are not modified by carbohydrate. The above results suggest that besides carbohydrate modifications there are other unknown factors like proper folding influencing the biological activities of FIPs. In this study, rFIP-nha was largely expressed in *E. coli* cells, GST-tag fusion simplified the purification procedure, GST-tag cutting off eliminated the effect on the protein folding, and lack of glycosylation didn’t influence the biological activities of rFIP-nha.

## 3. Experimental Section

### 3.1. Expression and Purification of rFIP-nha

#### 3.1.1. Gene Synthesis and Construction of the Expression Vector

After optimizing codons according to the *E. coli* preference, FIP-nha (GenBank ID: EEU37941.1) was synthesized by GenScript (Nanjing, China). Two flanking primers, FIP-nha-F (*Eco*RI 5'-GAATT CGCTAC TACCA ACG-3') and FIP-nha-R (*Xho*I 5'-CTCGA GTTAC TTCCA TTGGG CGAC-3'), were used to amplify the synthesized gene using La Taq polymerase (Takara BIO Inc., Dalian, China) for PCR. The amplified product was ligated into pMD18-T (Invitrogen, Carlsbad, CA, USA) and verified by sequencing. The gene was excised from pMD18-T-FIP-nha by *Eco*RI and *Xho*I, and sub cloned into the equivalent sites of the GST (glutathione-S-transferase) fusion vector pGEX-4T-1 (Amersham Pharmacia Biotech, Buckinghamshire, UK) to construct the recombinant plasmid pGEX-FIP-nha.

#### 3.1.2. Protein Expression and Purification

The plasmid pGEX-FIP-nha was transformed into *E. coli* Rosetta (Novagen, CA, USA) competent cells. Transformants were grown at 25 °C for 6 h with shaking at 250 rpm in 200 mL of LB medium containing 0.1 mM IPTG and 50 µg/mL ampicillin. Cells were harvested by centrifugation at 5000× *g* for 5 min, suspended in 10 mM PBS (10 mL, pH 7.2), and disrupted by sonication. The crude extract was applied to a GST column (Pharmacia, Uppsala, Sweden), equilibrated and eluted with PBS to remove contaminants. Bound protein was cleaved with thrombin (100 U/mL) at 30 °C for 24 h on the column, and cleaved FIP was finally eluted with PBS. Remaining un-cleaved fusion protein and GST were eluted with 10 mM glutathione. Thrombin was removed with Benzamidine sepharose 6B (GE Healthcare, Fairfield, CT, USA). Protein samples were quantified using a NanoDrop-1000 Spectrophotometer (Thermo Fisher Scientific, Waltham, MA, USA), and evaluated with Tricine-SDS-PAGE. In order to exclude the possibility of endotoxin contamination, rFIP-nha was examined by the Limulus Amoebocyte Lysate assay (LAL) (Associates of Cape Cod Inc., East Falmouth, MA, USA), employing the gel clot method (Supplementary Table S1).

#### 3.1.3. MALDI-MS Analysis

The protein band corresponding to rFIP-nha was excised from the Tricine-SDS-PAGE gel. Then the gel pieces were washed three times with ddH_2_O, decolorized with 50% acetonitrile/25 mM ammonium bicarbonate (100 µL, pH 8.0) for 15 min three times, followed by washing with ddH_2_O, dehydrating in 30 µL 100% acetonitrile for 5 min, and drying at 25 °C. The protein in the gel was digested overnight at 37 °C with 0.8 µg Trypsin Gold (Promega, Madison, WI, USA). The resulting peptides were subjected to MALDI-MS analysis using an ABI 4700 Proteomics Analyzer (Applied Biosystems, Foster City, CA, USA) at the Tianjin Biochip Corporation (Tianjin, China).

### 3.2. Hemagglutination Test

Hemagglutination activity of rFIP-nha was measured as described previously [24], using rabbit blood and all four human blood types (A, B, AB, and O). Cells were collected by centrifugation at 1000× *g* for 5 min, washed with PBS three times, and suspended in PBS at the concentration of 1.5% (*g*/*v*). Various concentrations of purified rFIP-nha (25 µL) were incubated with 25 µL of 1.5% blood solution in a 96-well V-bottom microplate at 25 °C for 2 h. Concanavalin A (ConA, 5 µg/mL) was used as a positive control.

### 3.3. The Immunomodulatory Activity Assay of rFIP-nha

#### 3.3.1. Mouse Splenocyte Proliferation Assay

The proliferation activity of rFIP-nha on mouse splenocytes was evaluated with the 3-(4,5-dimethylthiazol-2-yl)-2,5-diphenyl (MTT) method [45]. Six-week-old BALB/c mice were purchased from the Vital River Laboratory (Beijing, China) and splenocytes were isolated and suspended (1 × 10^5^ cell/mL) in RPMI1640 medium supplemented with 10% fetal calf serum, 100 U/mL penicillin and 100 µg/mL streptomycin. The cell suspension (100 µL) and rFIP-nha (1, 2, 4, 8, 16, and 32 µg/mL) were incubated in a 96-well microplate at 37 °C under 5% CO_2_ for 68 h, followed by addition of 20 µL MTT (5 mg/mL) and incubation for 4 h. ConA (5 µg/mL) and lipopolysaccharide (LPS, 2 µg/mL) were used as positive controls. After carefully removing the culture supernatant by aspiration, 100 µL DMSO was added to each well. The plate was gently shaken for 10 min and the absorbance of the mixture was measured at 570 nm with a Multiskan MK3 (Thermo Fisher Scientific, Waltham, MA, USA).

#### 3.3.2. IL-2 Release Assay

Splenocytes were prepared as described above and adjusted to 1 × 10^6^ cell/mL in complete RPMI1640 medium. One hundred microliter splenocytes and rFIP-nha (1, 2, 4, 8, 16, and 32 µg/mL) were seeded into a 96-well microplate and incubated at 37 °C under 5% CO_2_ for 48 h. Culture supernatant was collected by centrifugation at 1000× *g* for 5 min, and IL-2 in the supernatant was measured with an ELISA kit for mouse IL-2 (Uscn Life Science Inc., Wuhan, China). ConA (5 µg/mL) was used as a positive control.

### 3.4. The Antitumor Activity Assay of rFIP-nha

#### 3.4.1. Tumor Cell Proliferative Inhibitory Analysis

The inhibitory effects of rFIP-nha on gastric cancer MGC823 and liver cancer HepG2 cell lines were evaluated by counting viable cells with an MTT assay [25]. The cultured tumor cell lines, MGC823 and HepG2 (Cell Resource Center of Peking Union Medical College Hospital, Beijing, China), were suspended to 5 × 10^5^ cell/mL in complete DMEM and RPMI1640 medium, respectively. Aliquots of 100 µL cell suspension and 100 µL rFIP-nha (2, 4, 8, 16, 32, and 64 µg/mL) were seeded into a 96-well microplate. PBS was used as negative control. After cultivation for 24 h at 37 °C in a humidified 5% CO_2_ incubator, 20 µL MTT (5 mg/mL) was added and incubated for 4 h. After carefully removing the culture supernatant by aspiration, 100 µL DMSO was added to each well. The plate was gently shaken for 10 min. The percentage of viable cells was measured by MTT assay. Absorbance (OD) was measured at 570 nm using a Multiskan MK3. The IC_50_ value was determined as the concentration that caused 50% inhibition of cell proliferation [46].

#### 3.4.2. Tumor Cell Apoptosis Assay

The human leukemia HL60, gastric cancer MGC823 and liver cancer HepG2 cell lines (Cell Resource Center of Peking Union Medical College Hospital, Beijing, China) were suspended to 1 × 10^6^ cell/mL in complete RPMI1640 (HL60 and MGC823) and DMEM (HepG2) media. Then, 800 µL cell suspensions and 200 µL of various concentrations of rFIP-nha in PBS were incubated in a 24-well microplate at 37 °C under 5% CO_2_. RFIP-nha was used at 8, 16, and 32 µg/mL to treat HL60, MGC823 and HepG2 cells for 24 h. Untreated cells were used as negative controls. Cells were harvested by centrifugation at 1000× *g* for 5 min, washed twice with PBS and evaluated for apoptosis using an Annexin V-EGFP Apoptosis Detection Kit (Vigorous Biotechnology Beijing Co., Ltd., Beijing, China) and a BD FACSCalibur flow cytometer.

### 3.5. Statistical Analysis

All values are presented as means ± SD of three independent experiments performed in triplicate. Statistical comparisons were performed with one-way ANOVA using SPSS 19 (PASW statistics, IBM, New York, NY, USA). Differences were considered to be statistically significant at *p* < 0.05 and *p* < 0.01.

## 4. Conclusions

FIP-nha from *N. haematococca* was successfully expressed in *E. coli* expression system. The working condition for soluble rFIP-nha protein was at 25 °C induced with 0.1 mM IPTG for 6 h. The yield of purified rFIP-nha was up to 42.7 mg/L. Evaluation of biological activity of rFIP-nha indicated that this small peptide had a significant agglutinating activity with rabbit blood cells at concentrations greater than 1.275 µg/mL; maximally promoted splenocyte proliferation and induced IL-2 release at 2 μg/mL; as well as inhibited MGC-823 and HepG2 proliferation and induced HL60, MGC-823 and HepG2 apoptosis at different levels. Thus, rFIP-nha has the potential for therapeutic applications against human diseases.

## Supplementary Materials

Supplementary table can be found at http://www.mdpi.com/1422-0067/15/10/17751/s1.

## Supplementary Files

Supplementary File 1
