# Supplementary material for: Recombinant Expression of a Novel Fungal Immunomodulatory Protein with Human Tumor Cell Antiproliferative Activity from Nectria haematococca
Source: Int J Mol Sci. 2014 Sep 30;15(10):17751–64. doi: 10.3390/ijms151017751 (PMC4227187; doi:10.3390/ijms151017751)
Supplement: Supplementary File 1 [file ijms-15-17751-s001.pdf]

Supplementary Information

Table S1. LAL (Limulus amebocyte lysate) assay of endotoxin in rFIP-nha.

Samples	Clot	Results
Sterile water	−	negative
E. coli CSE (0.0075 EU/mL)	−	negative
E. coli CSE (0.015 EU/mL)	+	positive
E. coli CSE (0.03 EU/mL)	+	positive
E. coli CSE (0.06 EU/mL)	+	positive
PBS	−	negative
RFIP-nha (8 μg/mL)	−	negative
RFIP-nha (16 μg/mL)	−	negative
RFIP-nha (32 μg/mL)	−	negative

LAL assay was performed by gel-clot method in triplicate. *E. coli* Control Standard Endotoxin (CSE) were used as positive controls, and sterile water was used as a negative control. It was confirmed that the PBS and rFIP-nha samples were not diluted by endotoxin (LPS).
